# ID 120 - Avaliação de Incorporação da Rivastigmina para o Tratamento de Indivíduos com Doença de Parkinson e Demência no SUS

**DOI:** 10.5327/2237-9622.2025.v34s1.120

**Published:** 2025-11-25

**Authors:** Vinicius Lins Ferreira, Layssa Andrade Oliveira, Haliton Alves de Oliveira, Rosa Camila Lucchetta

**Keywords:** rivastigmina, protocolos clínicos, doença de Parkinson, demência

## Abstract

**Introdução:**

A doença de Parkinson (DP) é uma doença neurodegenerativa progressiva que se caracteriza pela degeneração e perda de neurônios dopaminérgicos. Estima-se que cerca de 30% dos pacientes apresentem demência relacionada à DP. Para estes pacientes não havia tratamento medicamentoso disponível no Sistema Único de Saúde (SUS), sendo a rivastigmina o único medicamento com registro na Agência Nacional de Vigilância Sanitária (Anvisa) para tal indicação. O objetivo deste estudo foi de avaliar a eficácia, a segurança, a custo-efetividade e o impacto orçamentário de rivastigmina cápsula e adesivo na perspectiva do SUS.

**Método:**

Uma revisão sistemática foi conduzida para avaliar as evidências da rivastigmina, de modo que foram realizadas buscas nas plataformas PubMed, Embase, LILACS e Cochrane Library. A avaliação do risco de viés foi realizada utilizando a ferramenta Risk of Bias (RoB) 2.0, e a qualidade das evidências foi graduada conforme o sistema GRADE. Além disso, uma análise de custo-efetividade (ACE: modelo de árvore de decisão; horizonte temporal de um ano; desfechos de efetividade: melhora clínica e anos de vida ajustado a qualidade [Avaq]; desfecho econômico: custos diretos médicos) e de impacto orçamentário (AIO) também foram desenvolvidas.

**Resultados:**

Apenas um ensaio clínico randomizado contemplou os critérios de elegibilidade e foi incluído, sendo que para função cognitiva avaliada pelo Miniexame do estado mental, observou-se melhora de 0,8 ponto no grupo tratado com rivastigmina + cuidado padrão e declínio de 0,2 ponto no grupo que recebeu placebo + cuidado padrão (DM: 1,0; p=0,03). Os resultados foram corroborados pela escala ADAS-Cog, em que houve redução dos escores no grupo rivastigmina e discreto aumento no grupo placebo, o que representa melhoria da função cognitiva para o primeiro grupo e declínio para o segundo (DM=2,90, p<0,001). No resultado do teste ADCS-CGIC, que traduz a gravidade global da doença, observou-se maior percentual de pacientes com melhora no grupo rivastigmina + cuidado padrão, comparado ao placebo + cuidado padrão (RR 1,37 [IC 95% 1,04-1,79]; p=0,02). Não foram identificadas diferenças entre os grupos para eventos adversos graves. A qualidade da evidência foi baixa para os desfechos de eficácia e moderada para os de segurança. Em relação à ACE, a utilização de rivastigmina (cápsulas) resultou em uma razão de custo-efetividade incremental de R$ 8 mil por paciente que apresentou melhora clínica, e de R$ 25 mil por Avaq. Ao considerar análises de outros cenários, observou-se que a utilização de rivastigmina em adesivo é custo-efetiva para o SUS (R$ 39 mil por AVAQ). A incorporação da rivastigmina elevaria o orçamento ao longo de cinco anos (cápsula ou adesivo, respectivamente, R$ 97.531.725 ou R$ 158.978.534,22; ambas as apresentações: R$ 131.941.938,13), considerando aproximadamente 30 mil de população elegível.

**Conclusão:**

Rivastigmina mostrou-se mais eficaz que o cuidado padrão, sem prejuízo da segurança, custo-efetiva e viável economicamente para o sistema. A tecnologia recebeu recomendação e decisão favorável à incorporação no SUS.

